# Dietary Capsaicin Improves Glucose Homeostasis and Alters the Gut Microbiota in Obese Diabetic ob/ob Mice

**DOI:** 10.3389/fphys.2017.00602

**Published:** 2017-08-25

**Authors:** Jun-Xian Song, Hui Ren, Yuan-Feng Gao, Chong-You Lee, Su-Fang Li, Feng Zhang, Long Li, Hong Chen

**Affiliations:** ^1^Department of Cardiology, Peking University People's Hospital Beijing, China; ^2^Beijing Key Laboratory of Early Prediction and Intervention of Acute Myocardial Infarction, Peking University People's Hospital Beijing, China; ^3^Center for Cardiovascular Translational Research, Peking University People's Hospital Beijing, China

**Keywords:** capsaicin, obesity, diabetes, glucose homeostasis, gut microbiota

## Abstract

**Background:** The effects of capsaicin on obesity and glucose homeostasis are still controversial and the mechanisms underlying these effects remain largely unknown. This study aimed to investigate the potential relationship between the regulation of obesity and glucose homeostasis by dietary capsaicin and the alterations of gut microbiota in obese diabetic ob/ob mice.

**Methods:** The ob/ob mice were subjected to a normal, low-capsaicin (0.01%), or high-capsaicin (0.02%) diet for 6 weeks, respectively. Obesity phenotypes, glucose homeostasis, the gut microbiota structure and composition, short-chain fatty acids, gastrointestinal hormones, and pro-inflammatory cytokines were measured.

**Results:** Both the low- and high-capsaicin diets failed to prevent the increase in body weight, adiposity index, and Lee's obesity index. However, dietary capsaicin at both the low and high doses significantly inhibited the increase of fasting blood glucose and insulin levels. These inhibitory effects were comparable between the two groups. Similarly, dietary capsaicin resulted in remarkable improvement in glucose and insulin tolerance. In addition, neither the low- nor high-capsaicin diet could alter the α-diversity and β-diversity of the gut microbiota. Taxonomy-based analysis showed that both the low- and high-capsaicin diets, acting in similar ways, significantly increased the Firmicutes/Bacteroidetes ratio at the phylum level as well as increased the *Roseburia* abundance and decreased the *Bacteroides* and *Parabacteroides* abundances at the genus level. Spearman's correlation analysis revealed that the *Roseburia* abundance was negatively while the *Bacteroides* and *Parabacteroides* abundances were positively correlated to the fasting blood glucose level and area under the curve by the oral glucose tolerance test. Finally, the low- and high-capsaicin diets significantly increased the fecal butyrate and plasma total GLP-1 levels, but decreased plasma total ghrelin, TNF-α, IL-1β, and IL-6 levels as compared with the normal diet.

**Conclusions:** The beneficial effects of dietary capsaicin on glucose homeostasis are likely associated with the alterations of specific bacteria at the genus level. These alterations in bacteria induced by dietary capsaicin contribute to improved glucose homeostasis through increasing short-chain fatty acids, regulating gastrointestinal hormones and inhibiting pro-inflammatory cytokines. However, our results should be interpreted cautiously due to the lower caloric intake at the initial stage after capsaicin diet administration.

## Introduction

The use of chili peppers as an integral part of the diet has a long history and is highly popular worldwide. Capsaicin is the major bioactive ingredient in chili peppers and plays an important role in a series of pathophysiological processes via activation of transient receptor potential vanilloid 1 (TRPV1) receptors. It has been reported that TRPV1 activation by capsaicin is involved in the regulations of metabolic disorders (Sun et al., 2016), cardiovascular disease (Sun et al., 2016), pain (O'Neill et al., 2012), cancer (Chapa-Oliver and Mejía-Teniente, 2016), respiratory disease (Banner et al., 2011), and dermatosis (Sharma et al., 2013). Among these regulations by dietary capsaicin, its beneficial effects on obesity-related disorders were extensively documented in experimental studies. In 2007, Zhang et al. found, for the first time, that dietary capsaicin protects mice against high-fat diet-induced adipogenesis and obesity by an increase in cytosolic calcium via activation of TRPV1 channels (Zhang et al., 2007). Similarly, activation of TRPV1 channels by dietary capsaicin has been reported to improve visceral fat remodeling through connexin-43-mediated Ca^2+^ influx (Chen et al., 2015). In addition, dietary capsaicin can alleviate obesity-related glucose intolerance by regulating inflammatory responses and fatty acid oxidation in obese mice fed with a high-fat diet (Kang et al., 2010). Accumulating evidence from human studies also has shown that ingestion of foods containing capsaicin is inversely associated with the prevalence of obesity and type 2 diabetes (Zsombok, 2013; Sun et al., 2016). However, Okumura et al. have reported that dietary capsaicin failed to reduce body weight gain and abdominal white adipose tissue accumulation in obese diabetic KKAy mice, although the blood glucose levels were significantly decreased (Okumura et al., 2012). In addition, by using a TRPV1 knockout mouse model, previous studies yielded seemingly contradictory findings that TRPV1 exerts protective (Lee et al., 2015), deleterious (Motter and Ahern, 2008), or no effects (Marshall et al., 2013) on high-fat diet-induced obesity. Therefore, the effects of dietary capsaicin on obesity and glucose homeostasis are still controversial, and the underlying mechanisms remain largely unknown.

The mammalian intestinal tract is colonized by a dense and complex community of commensal microorganisms referred to as the gut microbiota. The gut microbiota play a key role in many physiological and pathological events occurring in their hosts. The role of the gut microbiota in obesity was initially identified by the findings that conventionalization of adult germ-free mice with a normal microbiota harvested from the cecum of healthy mice led to an increase in body fat content and insulin resistance (Bäckhed et al., 2004). So far, mounting evidence indicates the involvement of the gut microbiota in the pathogenesis of obesity-related disorders through regulating energy metabolism (Bäckhed et al., 2004; Turnbaugh et al., 2006), fatty acid synthesis (Bäckhed et al., 2004, 2007; Hwang et al., 2015), gastrointestinal hormones production (Kimura et al., 2013), and low-grade inflammation (Cani et al., 2008; Vijay-Kumar et al., 2010; Fei and Zhao, 2013). In addition, the latest findings suggest that the gut microbiota represent a potential therapeutic avenue for obesity and other metabolic disorders. Accordingly, modifications of the gut microbiota by dietary interventions (Xiao et al., 2014), prebiotics or probiotics (Delzenne et al., 2011), medication (Shin et al., 2014), and genetic engineering (Chen et al., 2014) are effective strategies to inhibit the development of obesity-related disorders.

Although the existing findings emphasize the indispensable role of the gut microbiota in obesity and glucose metabolic disorders, whether the effects of dietary capsaicin on obesity and glucose homeostasis is mediated by the gut microbiota have not been fully identified. Therefore, this study aimed to investigate the potential relationship between the regulation of obesity and glucose homeostasis by dietary capsaicin and the alterations of gut microbiota in obese diabetic ob/ob mice.

## Methods

### Experimental animals

The experimental protocols were approved by the Institutional Animal Care and Use Committee of Peking University People's Hospital and were in adherence with the Guide for the Care and Use of Laboratory Animals published by the US National Institutes of Health (NIH Publication No. 85-23, revised 1996). All efforts were made to minimize animal suffering or discomfort and reduce the number of animals used.

Male obese diabetic ob/ob mice with a C57BL/6J background at 4–5 weeks of age were obtained from the Institute of Laboratory Animal Science, Chinese Academy of Medical Sciences & Peking Union Medical College. The mice were single-housed to prevent cross contamination of gut microbiota in specific pathogen-free animal facilities (maintained at 20–25°C, 50–55% relative humidity, and a 12/12-h light/dark cycle). They were provided with access to a laboratory diet and tap water *ad libitum*. All mice were allowed to acclimate for at least 1 week before the experiments.

### Experimental groups and diet

The ob/ob mice were randomized into the following three groups: (1) Normal diet group (*n* = 5): the mice received standard laboratory chow (Institute of Laboratory Animal Science, Chinese Academy of Medical Sciences & Peking Union Medical College); (2) Low-capsaicin diet group (*n* = 5): the mice received standard laboratory chow plus 0.01% capsaicin (Sigma-Aldrich, St. Louis, MO, USA); (3) High-capsaicin diet group (*n* = 5): the mice received standard laboratory chow plus 0.02% capsaicin (Sigma-Aldrich). All mice were fed for 6 weeks, and food intake was measured every week. The normal diet had 22.47% of kilocalories from protein, 12.11% of kilocalories from fat, and 65.42% of kilocalories from carbohydrate, with total energy content of 3.42 kcal/g. The ingredients of the diet were crude protein 19.2%, crude fat 4.6%, crude fiber 4.0%, crude ash 6.3%, moisture 8.8%, calcium 1.19%, phosphorus 0.87%, lysine 11.1 g/kg, methionine 4.5 g/kg, cystine 6.4 g/kg, nitrogen free extract 55.9%. The low-capsaicin diet and high-capsaicin diet were generated by adding 0.01 and 0.02% capsaicin to the normal diet, respectively.

### Measurement of obesity parameters

The obesity parameters including body weight, adiposity index, and Lee's obesity index were measured in all mice. The adiposity index was calculated by the formula [100 × (mesenteric fat weight + epididymal fat weight + perirenal fat weight) / body weight]; and Lee's obesity index was calculated by the formula [body weight (g)^0.33^ × 10^4^ / naso-anal length (mm)].

### Biochemical analyses

The fasting blood glucose levels were measured using an Accu-Chek Active blood glucose meter (Roche Diagnostics, Mannheim, Germany) by puncturing the tail vein. In addition, blood samples were collected from the retro-orbital plexus for determination of plasma insulin using a commercially available RIA kit (Beijing Furui Biotechnology Co., Ltd., China), according to the manufacturer's instructions.

### Oral glucose tolerance test (OGTT) and insulin tolerance test (ITT)

For the OGTT, the mice were administered with an oral glucose load of 2 g/kg body weight after 16 h of fasting. Before snips, the tail ends of rats were dipped into Bupivicaine (0.25%) for local anesthesia to reduce pain. The blood glucose levels were assessed using samples obtained from the tail vein at 0, 15, 30, 60, 90, and 120 min following the glucose load. The ITT was performed using a single intraperitoneal injection of insulin (0.75 IU/kg body weight) after 6 h of fasting. Blood samples were taken from the tail veins at 0, 15, 30, 60, 90, and 120 min after the insulin injection. Blood glucose concentrations were measured with a glucose analyzer as described above. The area under the curve (AUC) for each OGTT and ITT was calculated through trapezoidal approximation using GraphPad Prism software (version 6.0, GraphPad Software Inc., La Jolla, CA, USA) and expressed as a percentage of the normal diet group (100%).

### Pyrosequencing of the V4 region of 16S rRNA genes

Total DNA of gut microbiota was isolated from fresh fecal samples using a FastDNA Spin Kit For Feces (MP). The V4 hypervariable region of 16S rRNA genes was amplified with bar-coded primers (Forward 515 F: GTGCCAGCMGCCGCGGTAA; Reverse 806 R: GGACTACHVGGGTWTCTAAT) in a polymerase chain reaction (PCR) system thermocycler (ABI GeneAmp® 9700 system, Applied Biosystems, Foster City, CA, USA) using the following reaction conditions: initial denaturation at 95°C for 5 min, followed by 30 cycles of 95°C for 30 s (denaturation), 58°C for 30 s (annealing), and 72°C for 25 s (elongation), with a final extension at 72°C for 7 min. The products from different samples were mixed at equal ratios for sequencing using the Illumina HISeq 2500 platform.

### Bioinformatics analyses of microbiome data

Sequence data were analyzed using a combination of the software programs UPARSE (usearch version 8.0.1517), QIIME (version 1.9.1), and R (version 3.2.3). The poor-quality raw 16S rRNA reads were filtered out and trimmed by Trimmomatic (version 0.36) with default parameters. The pair-end reads were merged by PandaSeq (version 2.8; parameter: -t 0.90). All demultiplexed reads were clustered into operational taxonomic units (OTUs) at 97% sequence identity using the UPARSE pipeline. The OTU representative sequences were aligned against to the greengenes reference template set based on PyNAST (version 1.2.1). The phylogenetic tree was constructed using FastTree (version 2.1.3) with the filtered alignment. The Ribosomal Database Project (RDP) Classifier (version 2.2) was employed for taxonomy assignment against RDP 16S rRNA training set 9 with a confidence score ≥0.8. For the alpha-diversity metrics, richness estimators (e.g., ACE, Chao1) and diversity indices (e.g., Shannon index, Simpson index) were calculated, and rarefaction plots were generated with iterations of 10 at each sampling depth of 100 and increments of 100. For the beta-diversity metrics, the unweighted and weighted UniFrac distance matrices were calculated and visualized with Principal Coordinate Analysis (PCoA) analyses in QIIME.

### Measurement of short-chain fatty acids

Fecal samples from each mouse were suspended and homogenized in 1 mL deionized water. The pH value of the suspension was adjusted to 2–3 by adding 5 M hydrochloric acid, and then kept at room temperature for 10 min with intermittent shaking. After transferring into a polypropylene tube, the suspension was centrifuged at 5,000 rpm for 20 min. 2-ethylbutyric acid, which served as the internal standard, was added into the supernatant at a final concentration of 1 mM. The short-chain fatty acids was measured on an Agilent 7890A/5975C GC system (Agilent Technologies, PA, USA) according to the protocol previously described (Zhao et al., 2006). The levels of short-chain fatty acids were corrected by the wet weight of fecal sample.

### Measurement of plasma gastrointestinal hormones and pro-inflammatory cytokines

Plasma samples were obtained from the whole blood collected after 6 weeks of capsaicin feeding and stored at −80°C for further analysis. Levels of total GLP-1 (7–36 and 9–36) (Millipore), total ghrelin (Millipore), TNF-α (R&D Systems), IL-1β (R&D Systems) and IL-6 (R&D Systems) were measured by enzyme-linked immunosorbent assay according to manufacturer's instructions.

### Statistical analyses

Statistical analyses and graphing were performed using GraphPad Prism software (version 6.0). QIIME, STAMP, and R packages were used for analyzing or graphing the gut microbiota profiles. Quantitative data were expressed as means ± standard deviations (SD) or whisker box plots. Differences among groups for statistical significance were determined using one-way analysis of variance (ANOVA) followed by Tukey's *post hoc* test or the Kruskal-Wallis test. Correlations between bacterial abundance and glucose parameters were assessed by Spearman's correlation analysis. A *P*-value < 0.05 was considered statistically significant.

## Results

### Effects of dietary capsaicin on obesity parameters

**Figure 1:**
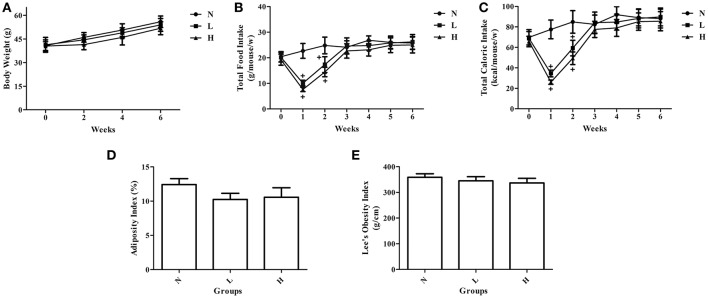
Effects of dietary capsaicin on obesity parameters. **(A)** Body weight; **(B)** total food intake, expressed as grams per mouse per week; **(C)** total caloric intake, expressed as kcal per mouse per week; **(D)** adiposity index, calculated according to the following formula: 100× (mesenteric fat weight + epididymal fat weight + perirenal fat weight)/body weight; **(E)** Lee's obesity index, calculated according to the following formula: body weight (g)^0.33^ × 10^4^/naso-anal length (mm). N, normal diet group (*n* = 5); L, low-capsaicin diet group (*n* = 5); H, high-capsaicin diet group (*n* = 5). Data are shown as the mean ± SD; ^+^*P* < 0.05, analyzed by one-way ANOVA with Tukey's *post hoc* test.

To investigate whether dietary capsaicin can prevent obesity, ob/ob mice were fed with a standard chow diet plus different doses of capsaicin for 6 weeks. The body weights of ob/ob mice in the normal diet group were gradually increased with time. However, neither the low-capsaicin diet nor the high-capsaicin diet was capable of preventing the increase of body weight in ob/ob mice during the 6-week study (Figure 1A). Similarly, dietary capsaicin at either the low or high dose also failed to inhibit the development of obesity, as evidenced by a similar adiposity index and Lee's obesity index when compared with the normal diet at 6 weeks (*P* > 0.05, Figures 1D,E). In addition, although both the low- and high-capsaicin diets led to the marked decreases in food intake and caloric intakes during the first 2 weeks compared with the normal diet (*P* < 0.05), the food and caloric intakes were not significantly different among the three groups from 3 to 6 weeks (*P* > 0.05, Figures 1B,C).

### Effects of dietary capsaicin on blood glucose and insulin levels

**Figure 2:**
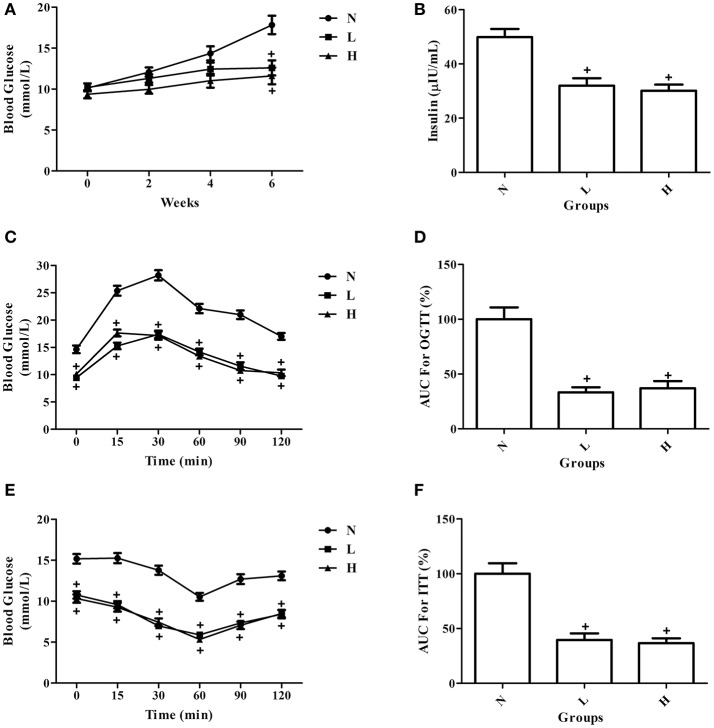
Effect of dietary capsaicin on glucose homeostasis. **(A)** Blood glucose; **(B)** insulin; **(C)** oral glucose tolerance test (OGTT); **(D)** area under the curve (AUC) for the OGTT, expressed as a percentage of the normal diet group (%); **(E)** insulin tolerance test (ITT); **(F)** area under the curve (AUC) for the ITT, expressed as a percentage of the normal diet group (%). N, normal diet group (*n* = 5); L, low-capsaicin diet group (*n* = 5); H, high-capsaicin diet group (*n* = 5). Data are shown as the mean ± SD; ^+^*P* < 0.05, analyzed by one-way ANOVA with Tukey's *post hoc* test.

To investigate the effect of dietary capsaicin on glucose homeostasis, fasting blood glucose and insulin levels were first determined in ob/ob mice. In agreement with the changes in body weight, the fasting blood glucose levels were increased gradually with time in ob/ob mice fed with a normal diet. Both the low-capsaicin diet and the high-capsaicin diet significantly inhibited the increase of fasting blood glucose and insulin levels at 6 weeks of feeding, and the inhibitory effects were comparable between the low-capsaicin and high-capsaicin diet groups (*P* > 0.05, Figures 2A,B).

### Effects of dietary capsaicin on glucose tolerance and insulin tolerance

To further assess the role of dietary capsaicin in regulating glucose homeostasis, the OGTT and ITT were performed in ob/ob mice at 6 weeks. The OGTT showed that the blood glucose levels were significantly lower in ob/ob mice fed with a capsaicin diet than in those with a normal diet at all-time points after glucose load (*P* < 0.05, Figure 2C). In addition, the AUC data for the OGTT indicated that dietary capsaicin resulted in a significantly reduced AUC in ob/ob mice fed with a capsaicin diet at either a low or a high dose than in those with a normal diet (*P* < 0.05), indicating a markedly improved glucose tolerance in capsaicin-treated diabetic mice (Figure 2D). However, no significant difference was observed in the improvement of glucose tolerance between the low-capsaicin and high-capsaicin diet groups (Figures 2C,D). Similar results reflecting the beneficial effect of dietary capsaicin on glucose homeostasis were also found with the ITT (Figures 2E,F).

### Effect of dietary capsaicin on the gut microbiota structure

**Figure 3:**
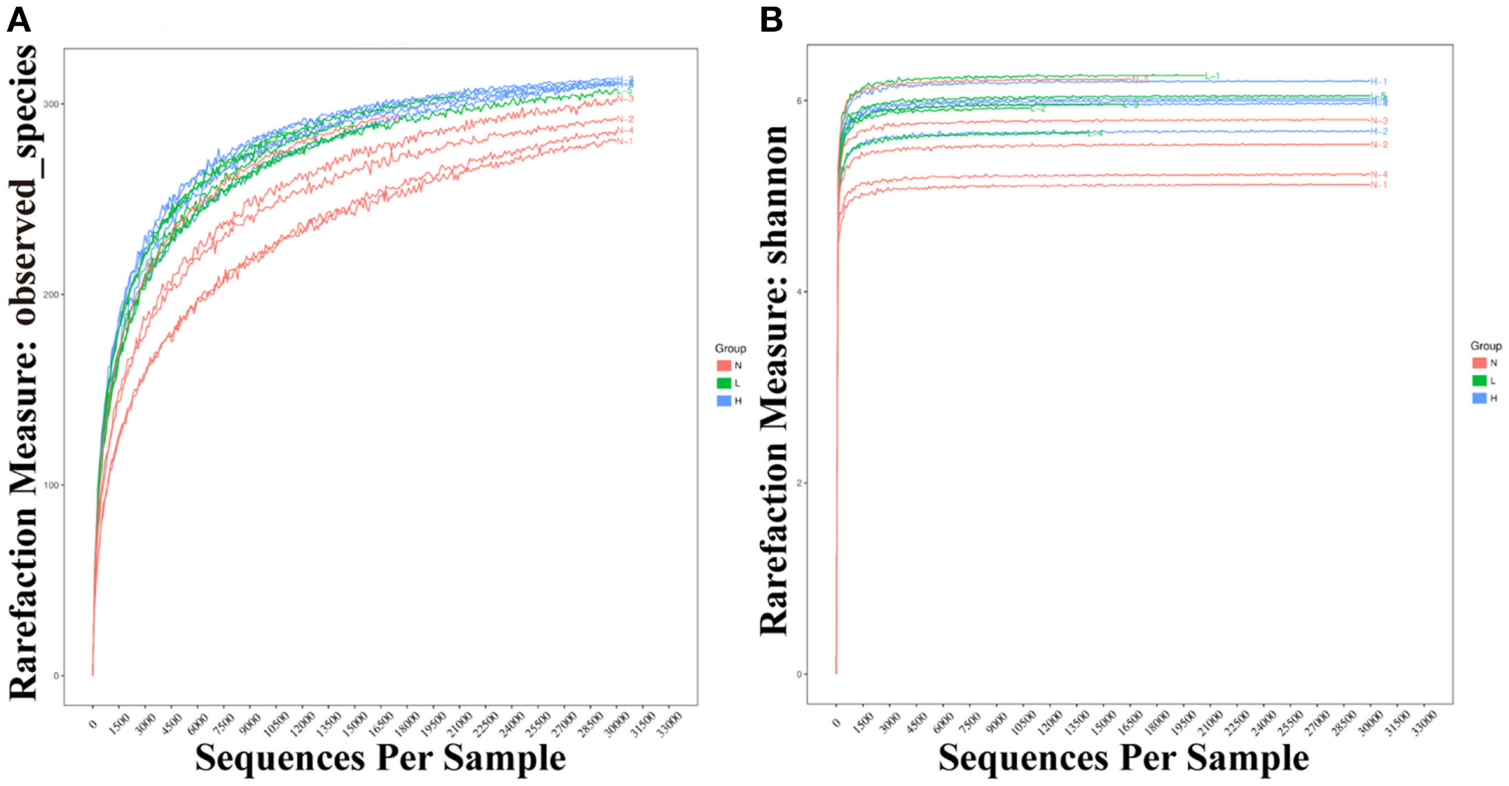
Rarefaction curves of observed species and Shannon-Wiener diversity for all samples. **(A)** Rarefaction curves of observed species from fecal samples of individual mice fed with a normal diet (red), low-capsaicin diet (green), or high-capsaicin diet (blue). **(B)** Rarefaction curves of Shannon-Wiener diversity from fecal samples of individual mice fed with a normal diet (red), low-capsaicin diet (green), or high-capsaicin diet (blue).

To investigate whether dietary capsaicin can lead to specific alterations in the gut microbiota structure in ob/ob mice, the fecal samples at 6 weeks after feeding were analyzed. A total of 792,163 high-quality sequences were obtained from 15 fecal samples (*n* = 5 per group, mean 52,811 ± 25,356 sequences per sample, range 17,467−103,159) by high-throughput pyrosequencing. The high quality sequences were then delineated into 337 OTUs at a similarity cut-off of 97%. The observed species and Shannon-Wiener diversity rarefaction curves reached the saturation phase (Figure 3), showing that the sequence depth obtained was adequate for all samples, although additional new phylotypes would possibly be identified by further sequencing.

**Figure 4:**
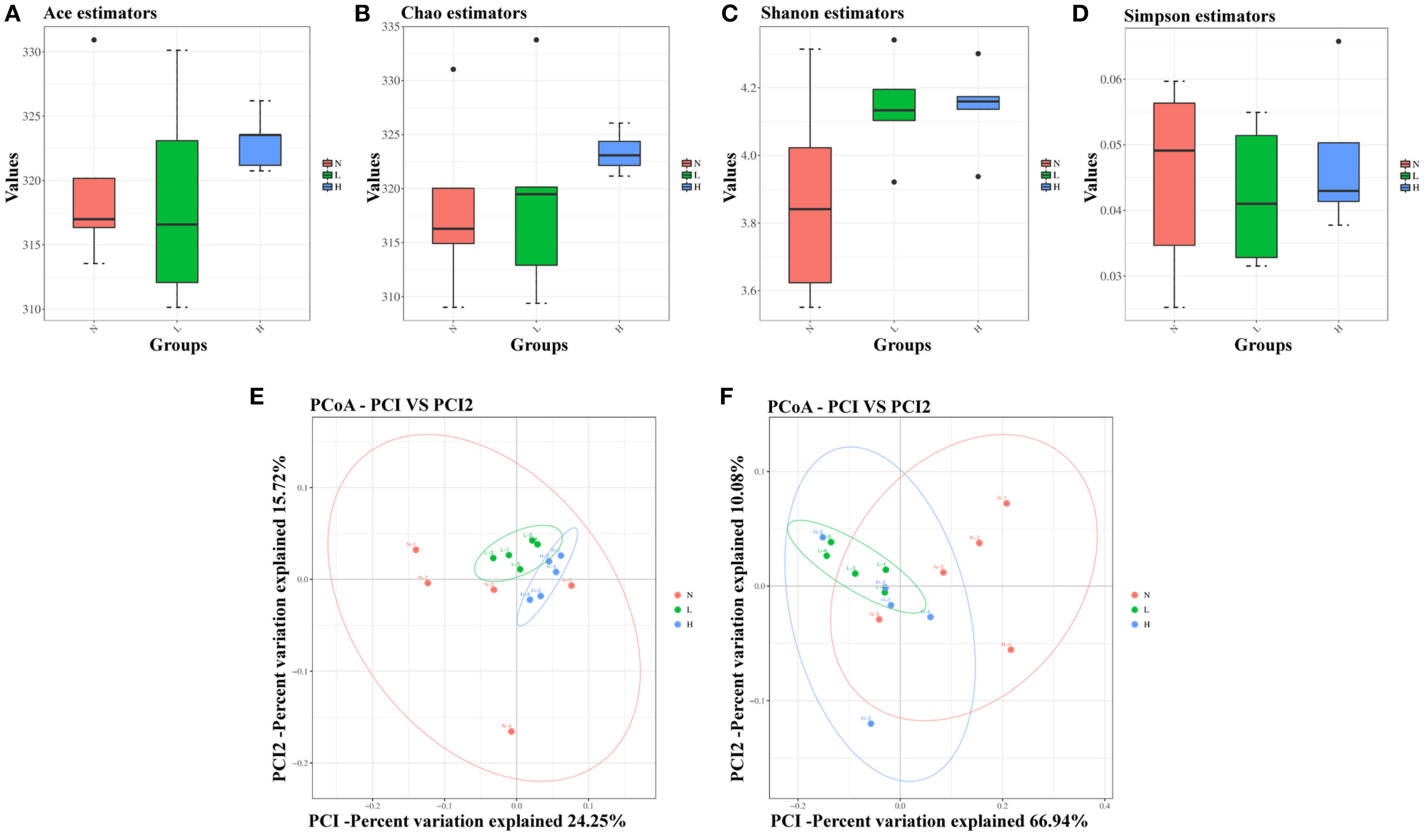
Effect of dietary capsaicin on the gut microbiota structure. **(A)** Ace estimator, **(B)** Chao estimator, **(C)** Shannon index, **(D)** Simpson index, **(E)** unweighted UniFrac distance-based principal coordinate analysis (PCoA), **(F)** weighted PCoA. N, normal diet group (*n* = 5); L, low-capsaicin diet group (*n* = 5); H, high-capsaicin diet group (*n* = 5). Data are shown as box and whisker plots. The box indicates the interquartile range (IQR, 75th to 25th percentiles of the data), and the mean value is shown as a line within the box; whiskers extend to the most extreme value within 1.5 × IQR, and outliers are shown as black dots. The results were analyzed by one-way ANOVA with Tukey's *post hoc* test or the Kruskal–Wallis test.

First, α-diversity analysis, which consisted of community richness and diversity (richness and evenness), was performed among the three groups at 6 weeks. No significant differences were detected in the richness (represented by the Ace and Chao estimator) or diversity (represented by the Shannon and Simpson index) among the three groups (Figures 4A–D). These results indicated that both the low- and high-capsaicin diets failed to alter the α-diversity. Next, β-diversity analysis was performed based on the unweighted and weighted UniFrac distance-based PCoA. Similarly, no separate clustering pattern among the three groups was found in relation to the low- and high-capsaicin diets by either unweighted or weighted UniFrac PCoA analysis (Figures 4E,F). These results indicated that the overall gut microbiota structure of ob/ob mice remained stable during dietary capsaicin intervention.

### Effect of dietary capsaicin on the gut microbiota composition

**Figure 5:**
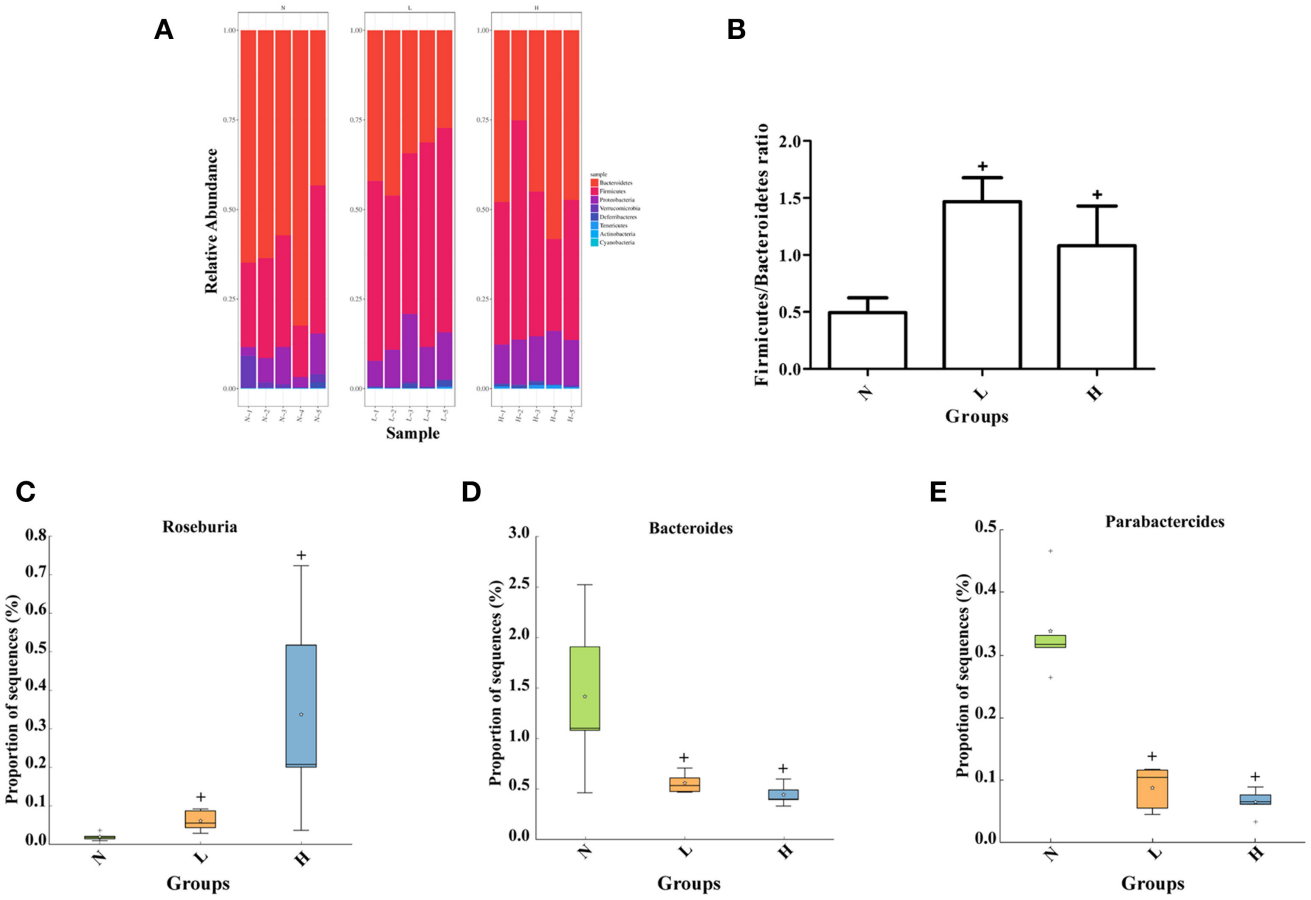
Effect of dietary capsaicin on the gut microbiota composition. **(A)** Changes in the taxonomic composition of the gut microbiota at the phylum level; **(B)** the Firmicutes/Bacteroidetes ratio; **(C)** the abundance of *Roseburia*; **(D)** the abundance of *Bacteroides*; **(E)** the abundance of *Parabacteroides*. N, normal diet group (*n* = 5); L, low-capsaicin diet group (*n* = 5); H, high-capsaicin diet group (*n* = 5). Data are shown as box and whisker plots. The box indicates the interquartile range (IQR, 75th to 25th percentiles of the data), and the mean value is shown as a line within the box; whiskers extend to the most extreme value within 1.5 × IQR, and outliers are shown as crosses. The results were analyzed by one-way ANOVA with Tukey's *post hoc* test or the Kruskal–Wallis test, ^+^*P* < 0.05.

To further determine the relationship between dietary capsaicin and the gut microbiota composition in the regulation of glucose homeostasis, taxonomy-based analysis was performed at the phylum and genus levels. At the phylum level, both the low- and high-capsaicin diets significantly increased the relative abundance of Firmicutes but decreased the relative abundance of Bacteroidetes. Accordingly, the ratio between Firmicutes and Bacteroidetes, a widely used marker of gut dysbiosis, was significantly higher in ob/ob mice subjected to dietary capsaicin intervention than a normal diet (Figures 5A,B). At the genus level, both low- and high-capsaicin-treated ob/ob mice showed enriching effects on *Roseburia* abundance and inhibiting effects on *Bacteroides* and *Parabacteroides* abundances (Figures 5C–E). In addition, the effects of dietary capsaicin on the gut microbiota composition at the phylum and genus levels showed no significant differences between the low-capsaicin and high-capsaicin diet groups (Figures 5B–E).

### Correlations between the glucose parameters and the gut microbiota abundance

**Figure 6:**
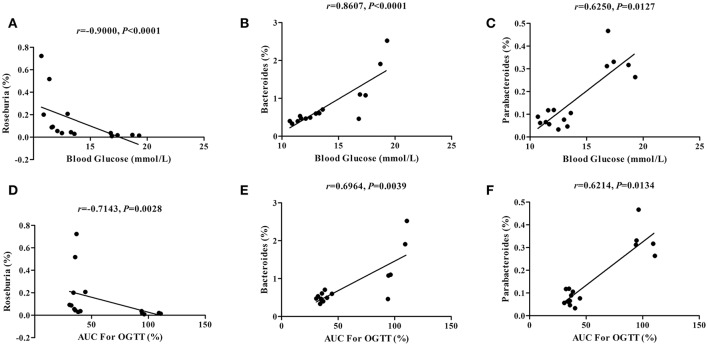
Correlations between the glucose parameters and the gut microbiota abundance. **(A–C)**: Correlations of the blood glucose level with the abundance of *Roseburia* **(A)**, *Bacteroides* **(B)**, and *Parabacteroides* **(C)**. **(D–F)** Correlations of the area under the curve (AUC) for the oral glucose tolerance test (OGTT) with the abundance of *Roseburia* **(D)**, *Bacteroides* **(E)**, and *Parabacteroides* **(F)**. The data were analyzed by Spearman correlation analysis.

To assess whether dietary capsaicin-induced glucose homeostasis improvement is associated with alterations of the gut microbiota in ob/ob mice, Spearman correlation analysis was performed to determine the correlations between the glucose parameters and the bacterial abundance at the genus level. The analysis revealed a strong negative correlation between the fasting blood glucose level and the abundance of *Roseburia* (*r* = −0.9000, *P* < 0.0001), and positive correlations between the fasting blood glucose level and the abundances of *Bacteroides* (*r* = 0.8607, *P* < 0.0001) and *Parabacteroides* (*r* = 0.6250, *P* = 0.0127) (Figures 6A–C). In addition, similar findings were found in the correlations between the AUC of the OGTT and the abundances of *Roseburia* (*r* = −0.7143, *P* = 0.0028), *Bacteroides* (*r* = 0.6964, *P* = 0.0039), and *Parabacteroides* (*r* = 0.6214, *P* = 0.0134) (Figures 6D–F).

### Effects of dietary capsaicin on short-chain fatty acids, gastrointestinal hormones, and pro-inflammatory cytokines

**Figure 7:**
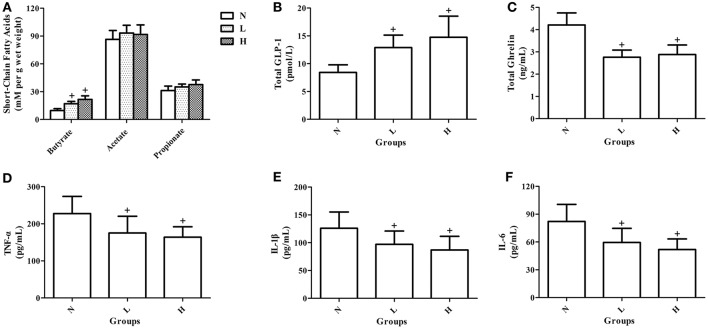
Effects of dietary capsaicin on short-chain fatty acids, gastrointestinal hormones, and pro-inflammatory cytokines. **(A)** Fecal short-chain fatty acid levels; **(B)** plasma total GLP-l level; **(C)** plasma total ghrelin level; **(D)** plasma TNF-α level; **(E)** plasma IL-1β level; **(F)** plasma IL-6 level. N, normal diet group (*n* = 5); L, low-capsaicin diet group (*n* = 5); H, high-capsaicin diet group (*n* = 5). Data are shown as the mean ± SD; ^+^*P* < 0.05. The data analyzed by one-way ANOVA with Tukey's *post hoc* test.

To observe the potential mechanisms underlying the improved glucose homeostasis by dietary capsaicin, the levels of fecal short-chain fatty acids, plasma gastrointestinal hormones and plasma pro-inflammatory cytokines were measured in ob/ob mice after 6 weeks of capsaicin feeding. Analysis of short-chain fatty acids showed that both the low- and high-capsaicin diets significantly increased the fecal butyrate level (*P* < 0.05, Figure 7A), but did not affect the fecal acetate and propionate levels (*P* > 0.05, Figure 7A), as compared with the normal diet. In addition, the plasma total GLP-1 level was higher, and the plasma total ghrelin, TNF-α, IL-1β, and IL-6 levels were lower in ob/ob mice fed with the low- or high- capsaicin diet than those with the normal diet (*P* < 0.05, Figures 7B–F).

## Discussion

Using a classic animal model simulating human obesity-related type 2 diabetes, this study provided novel evidence that dietary capsaicin significantly prevented the increases of fasting glucose and insulin as well as markedly alleviated impaired glucose tolerance and insulin resistance in obese diabetic ob/ob mice. However, it failed to affect the obesity phenotypes including body weight, Lee's obesity index, and the adiposity index. The mechanisms underlying these beneficial effects of dietary capsaicin on glucose homeostasis are likely associated with alterations in the gut microbiota composition, as evidenced by an increased abundance of *Roseburia* and a decreased abundances of *Bacteroides* and *Parabacteroides*. In addition, these altered bacteria at the genus level induced by dietary capsaicin contribute to improved glucose homeostasis by increasing short-chain fatty acids, regulating gastrointestinal hormones and inhibiting pro-inflammatory cytokines. To the best of our knowledge, these findings, for the first time, indicate the potential therapeutic role of dietary capsaicin in improving glucose homeostasis by regulating the gut microbiota in an obese diabetic animal model.

Numerous studies have investigated the effects of dietary capsaicin on obesity and glucose homeostasis. Among these studies, dietary capsaicin has been recognized to exert a beneficial effect on preventing obesity and to improve glucose homeostasis by activation of TRPV1 (Zhang et al., 2007; Kang et al., 2010; Chen et al., 2015). Similar results also have been found in high-fat diet-induced obese mice subjected to topical application of capsaicin cream (Lee et al., 2013) as well as in a Zucker diabetic fatty rat model of systemic sensory nerve desensitization by subcutaneous injection of high-dose capsaicin (Gram et al., 2005). However, our study showed that dietary capsaicin only improved glucose homeostasis but did not inhibit obesity-related phenotypes in obese diabetic ob/ob mice. Our results are consistent with the findings from a study performed in obese diabetic KKAy mice fed with capsaicin (Gram et al., 2005). The reasons that can account for these conflicting results are as follows: (1) Dietary capsaicin is likely to exert anti-obesity and antihyperglycemic effects in both dose- and time-dependent manners. A number of animal studies have indicated that the inhibitory effect of capsaicin on obesity relies on a sufficient dose exceeding 10 mg/kg body weight or 0.01% capsaicin in the diet and a lasting administration period of 5–6 weeks (Zhang et al., 2007; Kawabata et al., 2009; Kang et al., 2010; Okumura et al., 2012; Chen et al., 2015; Márquez-Ibarra et al., 2016). In addition, the capsaicin dose that can improve glucose homeostasis was lower than that that can inhibit obesity, and the improvement of glucose homeostasis occurs prior to the inhibition of obesity (Okumura et al., 2012). In this study, dietary capsaicin failed to improve obesity, probably due to the short capsaicin feeding time and/or the low capsaicin dose. (2) The anti-obesity and antihyperglycemic effects of dietary capsaicin may depend on the obese diabetic animal models used in the studies. According to previous evidence, the beneficial roles of dietary capsaicin in regulating obesity and glucose homeostasis have been consistently reported in high-fat diet-induced obesity models (Zhang et al., 2007; Kang et al., 2010; Chen et al., 2015). Nevertheless, our and another study (Okumura et al., 2012) were carried out in spontaneous obese diabetic models with genetic mutations (ob/ob mice or KKAy mice), which demonstrated that dietary capsaicin only improved glucose homeostasis without affecting the obesity-related phenotypes. Clearly, the pathogenesis and features of genetic diabetic models are substantially different from those of dietary models.

Numerous studies have demonstrated that many dietary components are able to inhibit obesity and/or improve glucose homeostasis by regulating the gut microbiota. However, few studies have reported the effects of dietary capsaicin on the gut microbiota in the presence of obesity. Baboota et al. found that the oral administration of capsaicin (2 mg/kg, po) for 3 months could inhibit obesity-related phenotypes by enriching the abundances of gut *Prevotella, Lactobacillus*, and *Akkermansia* in high-fat diet-fed mice, according to quantitative PCR data (Baboota et al., 2014). Moreover, Hochkogler et al. reported that a 12-week intervention with daily intake of 0.15 mg of nonivamide, a TRPV1 agonist, prevented dietary-induced body fat gain in moderately overweight subjects, but fecal microbiome read outs were not affected (Hochkogler et al., 2016). In addition, Kang et al. investigated the effects of both a low-capsaicin diet (5 mg/d) and a high-capsaicin diet (10 mg/d) for 2 weeks on the gut microbiota using 16S rRNA gene sequencing in healthy subjects. The results showed that dietary capsaicin did not affect the taxonomic α- and β-diversity but increased the Firmicutes/Bacteroidetes ratio and Faecalibacterium abundance; however, there were no changes in plasma glucose and insulin levels, body mass index, or the waist-hip ratio during the study period (Kang et al., 2016). Similarly, our study also showed that neither a low-capsaicin diet (0.01%) nor a high- capsaicin diet (0.02%) altered the α- and β-diversity; but capsaicin at both doses resulted in marked changes in the gut microbiota composition including an increased Firmicutes/Bacteroidetes ratio and *Roseburia* abundance as well as a decreased abundances of *Bacteroides* and *Parabacteroides*.

The Firmicutes/Bacteroidetes ratio in the stool is a gauge of the overall gut microbiota balance. It has been reported that an increase in the Firmicutes/Bacteroidetes ratio is associated with obesity and a worsened glucose tolerance. On the contrary, a growing number of studies have shown that no significant differences exist in the abundances of Firmicutes and Bacteroidetes between lean and obese individuals (Zhang et al., 2009; Schwiertz et al., 2010; Jumpertz et al., 2011). Also, Zhang *et al*. found that despite an increased Firmicutes/Bacteroidetes ratio, both berberine and metformin inhibited obesity and improved glucose homeostasis in high-fat diet-induced mice (Zhang X. et al., 2015). Therefore, whether the antihyperglycemic effect of dietary capsaicin contributes to an altered Firmicutes/Bacteroidetes ratio needs further investigation. In addition, previous studies have revealed that alterations of specific bacteria at the genus level are involved in the regulation of glucose homeostasis. The increased abundance of *Roseburia* (Neyrinck et al., 2012; Ryan et al., 2014) is positively correlated while the decreased abundances of *Bacteroides* (Dao et al., 2011; Dewulf et al., 2012; Ryan et al., 2014) and *Parabacteroides* (Dao et al., 2011) were negatively correlated with improved glucose homeostasis when subjected to definite interventions. The above-mentioned gut microbiota changes may increase the production of short-chain fatty acids and inhibit inflammatory responses in the gut. Thus, we believe that the beneficial effects of dietary capsaicin on glucose homeostasis are likely associated with specific microbial changes at the genus level. In contrast, another study found an opposite change in the abundances of *Bacteroides* and *Parabacteroides* at the genus level that contributed to improvement of glucose homeostasis (Sung et al., 2017). These conflicting findings in definite microbial changes could be attributed to different diet interventions, animal species, and/or diabetic models among the various studies.

We speculate that the mechanisms underlying the effect of dietary capsaicin on the gut microbiota composition involve the following aspects. The gut is extensively innervated by TRPV1-expressing primary afferent sensory nerves (Clapham, 2003; Nilius et al., 2007; Allais et al., 2017). TRPV1 is an important sensory transducer, which plays a key role in the regulation of intestinal tract function (Clapham, 2003; Nilius et al., 2007; Allais et al., 2017). In addition, activation of TRPV1 on the intestinal tract by dietary capsaicin leads to altered intestinal sensitivity and excitability as well as the local release of neuropeptides, including calcitonin gene-related peptide and substance P (Clapham, 2003; Nilius et al., 2007; Allais et al., 2017). Altered intestinal sensitivity and excitability are probably implicated in the maintenance of gut microbiota homeostasis (Wiles et al., 2016; Tap et al., 2017). Moreover, bacteria can sense specific neurotransmitters, neuropeptides, and neurohormones with their membrane proteins acting as specific sensors (Sperandio et al., 2003; Lyte, 2004; Holzer, 2016). The local release of neuropeptides can also regulate the structure and composition of the gut microbiota by changing the immune and inflammatory conditions in the intestinal tract. Indirect evidence also shows that both calcitonin gene-related peptide and substance P are crucial regulators of cutaneous microbiota homeostasis (N'Diaye et al., 2017).

A wealth of studies found that the regulation of gut microbiota on obesity-related disorders was related to short-chain fatty acid synthesis (Bäckhed et al., 2004, 2007; Hwang et al., 2015), gastrointestinal hormones production (Kimura et al., 2013) and systemic low-grade inflammation (Cani et al., 2008; Vijay-Kumar et al., 2010; Fei and Zhao, 2013). Our present study showed that dietary capsaicin led to the increase of *Roseburia* abundance and the decrease of *Bacteroides* and *Parabacteroides* abundances. In addition, dietary capsaicin also significantly increased the fecal butyrate and plasma total GLP-1 levels, but decreased plasma total ghrelin, TNF-α, IL-1β, and IL-6 levels. *Roseburia* serves as one of butyrate-producing bacteria species (Machiels et al., 2014; Zhang J. et al., 2015; Rivière et al., 2016), and butyrate promotes GLP-1 hormone secretion (Lin et al., 2012; Yadav et al., 2013) and suppresses systemic inflammation (Russo et al., 2012; Singh et al., 2014). Thus, we speculated that dietary capsaicin might induce some specific bacteria in obese diabetic ob/ob mice, which contributed to its antihyperglycemic effects by regulating short-chain fatty acids, gastrointestinal hormones and pro-inflammatory cytokines.

This study has some limitations. First, the results should be cautiously interpreted due to the relatively small sample size and short intervention period. Second, metagenomic analysis was not used to analyze the alterations in the gut microbiota structure and functions comprehensively. Third, fecal microbiota transplantation or depletion was not performed to further explore the role of the gut microbiota in dietary capsaicin-mediated glucose homeostasis improvement. Four, dietary capsaicin led to reduced caloric intake in the first 2 weeks as compared with normal diet due to the decrease of palatability, the difference in caloric intake was likely to be a potential confounder in our results.

## Conclusions

In summary, this study found that capsaicin intake led to significant improvement of glucose homeostasis in obese diabetic ob/ob mice, although it had no inhibitory effects on obesity-related phenotypes. The beneficial effect of dietary capsaicin on glucose homeostasis is likely related to the increased abundance of *Roseburia* and the decreased abundances of *Bacteroides* and *Parabacteroides* at the genus level, and which contribute to the improvement of glucose homeostasis by increasing short-chain fatty acids, regulating gastrointestinal hormones and inhibiting pro-inflammatory cytokines. These results offer a novel insight that alterations in the gut microbiota composition may be the potential mechanism underlying the antihyperglycemic effect of dietary capsaicin. However, our results should be interpreted cautiously due to the lower caloric intake at the initial stage after capsaicin diet administration.

## Author contributions

HC conceived and designed the experiments; JS, HR, YG, and CL performed the experiments, JS, SL, FZ, and LL analyzed the data; JS and HC wrote the paper.

### Conflict of interest statement

The authors declare that the research was conducted in the absence of any commercial or financial relationships that could be construed as a potential conflict of interest.
